# Bioethanol Potential of Energy Sorghum Grown on Marginal and Arable Lands

**DOI:** 10.3389/fpls.2018.00440

**Published:** 2018-04-09

**Authors:** Chaochen Tang, Songbo Li, Meng Li, Guang H. Xie

**Affiliations:** ^1^College of Agronomy and Biotechnology, China Agricultural University, Beijing, China; ^2^National Energy R&D Center for Non-food Biomass, China Agricultural University, Beijing, China

**Keywords:** saline-land, dry wasteland, biomass sorghum, sweet sorghum, theoretical ethanol yield

## Abstract

Field experiments were conducted in marginal lands, i.e., sub-humid climate and saline-land (SHS) and semi-arid climate and wasteland (SAW), to evaluate ethanol potential based on the biomass yield and chemical composition of biomass type (var. GN-2, GN-4, and GN-10) and sweet type (var. GT-3 and GT-7) hybrids of energy sorghum [*Sorghum bicolor* (L.) Moench] in comparison with sub-humid climate and cropland (SHC) in northern China. Results showed that environment significantly (*p* < 0.05) influenced plant growth, biomass yield and components, and subsequently the ethanol potential of energy sorghum. Biomass and theoretical ethanol yield of the crop grown at SHS (12.2 t ha^−1^ and 3,425 L ha^−1^, respectively) and SAW (8.6 t ha^−1^ and 2,091 L ha^−1^, respectively) were both statistically (*p* < 0.001) lower than values at the SHC site (32.6 t ha^−1^ and 11,853 L ha^−1^, respectively). Higher desirable contents of soluble sugar, cellulose, and hemicellulose were observed at SHS and SHC sites, while sorghum grown at SAW possessed higher lignin and ash contents. Biomass type sorghum was superior to sweet type as non-food ethanol feedstock. In particular, biomass type hybrid GN-10 achieved the highest biomass (17.4 t ha^−1^) and theoretical ethanol yields (5,423 L ha^−1^) after averaging data for all environmental sites. The most productive hybrid, biomass type GN-4, exhibited biomass and theoretical ethanol yields >42.1 t ha^−1^ and 14,913 L ha^−1^, respectively, at the cropland SHC site. In conclusion, energy sorghum grown on marginal lands showed a very lower ethanol potential, indicating a considerable lower possibility for being used as commercial feedstock supply when compared with that grown on regular croplands. Moreover, screening suitable varieties may improve energy sorghum growth and chemical properties for ethanol production on marginal lands.

## Introduction

Industrial-scale cultivation of non-food energy crops for biofuels production is generally recognized as a positive step toward preventing energy shortages and decreasing greenhouse gas emissions (Qin et al., 2011; Sanscartier et al., 2014). As part of China's comprehensive energy plan, its bioenergy industry is vigorously accelerating cellulosic ethanol fuel production and diversifying feedstock supplies to include new crops such as cassava and sweet sorghum. In 2020, ethanol yield will reach 4.0 million tons, a 90% increase from 2.1 million tons in 2015, according to *the 13th 5-Year Plan for bioenergy development* released by the National Energy Administration of China. Due to China's fairly limited cultivatable land resources, national policy has implemented land-use planning. As part of the overall plan, biofuel feedstock production will be limited to marginal lands to avoid land-use competition with food crops to maintain greater food security (Zhuang et al., 2011).

Energy sorghum, including biomass and sweet type varieties, has recently gained favor as bioethanol feedstock amongst numerous candidate crops (Rooney et al., 2007; Tew et al., 2008; Xie, 2012). Low input requirements, wide adaptability, and remarkable biological productivity confer better energy balance to sorghum as compared to other competing crops (Yu et al., 2008). Using current renewable energy technologies, soluble sugars and structural carbon compounds (cellulose and hemicellulose) in energy sorghum stems and leaves could be the most promising approach for the first and second generation ethanol production (Zhao et al., 2009; Zegada-Lizarazu and Monti, 2012; Cotton et al., 2013). Thus, knowledge of energy sorghum biomass chemical composition is a prerequisite for effective industrial production because composition directly impacts performance in various energy conversion processes. For example, cellulosic biomass is optimally converted to ethanol when lignin content is low (Weng et al., 2008). Lignin cannot be converted into carbohydrates and exerts a recalcitrant effect on conversion (Rocateli et al., 2012). In addition, high ash content may reduce efficiency of thermochemical conversion of biomass to fuel (Cassida et al., 2005).

The impact of environment factors including land type should be considered to select biomass feedstock crops and varieties. Rocateli et al. (2012) evaluated three types of sorghum (grain, forage, and photoperiod-sensitive sorghum) grown in the southern U.S. and observed that environment and genotype both exerted sizeable effects on biomass yield and chemical composition. Performances of biomass yield and its components of energy sorghum have been well documented by previous reports on the basis of its production on arable land (Amaducci et al., 2004; Tew et al., 2008; Zhao et al., 2009, 2012; Maw et al., 2016; Pannacci and Bartolini, 2016).

However, sorghum is particularly well-adapted to marginal land and constraints conditions, such as water deficits, salinity, and alkalinity (Dalla Marta et al., 2014; Regassa and Wortmann, 2014; Schmer et al., 2014). Sweet sorghum provided sufficient total sugar and ethanol yields in fields with a saline soil, even if it received 50–75% of the irrigation water typically applied to sorghum in Northern Greece (Vasilakoglou et al., 2011). On dryland in Nebraska one sweet sorghum cultivar was found to be competitive with grain crops for some biofuel criteria, but it was not competitive with grain crops for total or net liquid transportation fuel produced per hectare (Wortmann et al., 2010). Sweet sorghum exhibited a better energy efficiency (Ren et al., 2012) and economic return (Liu et al., 2015) to scale on investment than cotton or sunflower did on saline-alkali land in northern China. According to an industrial survey, the non-food feedstock cost was found to be 70–80% of the total ethanol production cost (Xie, 2012). Crop production in marginal lands faces a lack of infrastructural conditions and lower soil fertility, resulting in a higher feedstock cost than the same crop grown in regular croplands. However, previous reports comparing biomass yield and chemical composition of energy sorghum grown in marginal and croplands do not exist. Moreover, previous studies focused on sweet sorghum and few data are available on biomass sorghum, which has been recognized as a promising feedstock type for cellulosic ethanol production.

Therefore, the objectives of this study were: (1) to compare the variation in calculated ethanol potential based on biomass yield and chemical composition of energy sorghum grown on marginal and arable cropland under different climatic conditions; (2) to clarify the difference in biomass yield and chemical composition between biomass and sweet sorghum; and (3) to screen for suitable energy sorghum hybrids which could achieve high biomass yield and quality under marginal and arable land conditions for maximal ethanol production in northern China. The expected findings of this work could be helpful to evaluate the possibility of growing energy sorghum on marginal lands for commercial ethanol production in northern China.

## Materials and methods

### Site description

Field experiments were conducted in northern China at three different sites with distinct environmental characteristics, i.e., sub-humid climate and saline-land (SHS), semi-arid climate and wasteland (SAW), and sub-humid climate and cropland (SHC) (Table 1). These locations were selected based on the results of Zhang et al. (2010), who reported that Inner Mongolia ranks the highest for ethanol production potential from sweet sorghum, followed by Hebei and next by the northern Shandong Province. Thus, these regions should be regarded as priority regions for energy sorghum based biofuel feedstock production in northern China. Soil samples at a depth of 0–30 cm were collected before sowing in order to determine the main soil physical and chemical properties (Table 2). Weather data for the three sites during the energy sorghum growth period were also collected from nearby meteorological stations.

**Table 1:** Description of the study sites of sub-humid climate and saline-land (SHS), semi-arid climate and wasteland (SAW), and sub-humid climate and cropland (SHC).

**Parameter**	**SHS site**	**SAW site**	**SHC site**
Location	Binzhou, Shandong	Ordos, Inner Mongolia	Zhuozhou, Hebei
Latitude	37°42′N	39°10′N	39°28′N
Longitude	118°17′E	109°53′E	115°51′E
Elevation (m)	8	1,032	42
Climate type	Sub-humid	Semi-arid	Sub-humid
Land type	Saline-land	Wasteland	Cropland
Multi-year mean yearly precipitation (mm)	563	368	576
Multi-year mean yearly potential evaporation (mm)	1,213	2,506	1,575
Multi-year mean yearly average temperature (°C)	13	7	12
Multi-year mean yearly maximum temperature (°C)	19	12	19
Multi-year mean yearly minimum temperature (°C)	8	2	9

**Table 2:** Main soil properties and meteorological characteristics during the growth period of energy sorghum at the experimental sites of sub-humid climate and saline-land (SHS), semi-arid climate and wasteland (SAW), and sub-humid climate and cropland (SHC).

**Parameter**	**SHS site**	**SAW site**		**SHC site**
	**2013**	**2013**	**2014**	**2014**
Sand^a^ (%)	66.3	91.8	86.1	67.1
Silt^a^ (%)	31.0	7.6	13.1	32.1
Clay^a^ (%)	2.7	0.6	0.8	0.8
pH	8.2	8.0	8.1	7.9
Total salinity (g kg^−1^)	7.8	2.1	2.0	0.6
Soil organic matter (g kg^−1^)	5.4	0.7	0.7	12.0
Total nitrogen (g kg^−1^)	0.4	0.5	0.6	0.7
Available phosphorus (mg kg^−1^)	4.6	6.1	6.2	21.6
Available potassium (mg kg^−1^)	194.6	54.1	55.3	75.4
Rainfall (mm)	649.0	429.5	375.6	390.4
Relative humidity (%)	70.1	50.9	51.9	68.3
Daily mean temperature (°C)	24.4	18.8	18.9	23.0
Sunshine hours (h)	1044.0	1309.1	1163.4	1008.5
Solar radiation (MJ m^−2^)	2729.7	3029.5	3450.8	3013.2
Accumulated temperature (≥13°C)	1717.0	863.5	799.2	1704.3
Diurnal temperature difference (°C)	9.7	9.9	9.7	11.3

a *The soil texture was defined as sand, 0.02–2.0 mm; silt, 0.002–0.02 mm, and clay, < 0.002 mm*.

### Experimental design and operation

Five energy sorghum hybrids including biomass type (var. GN-2, GN-4, and GN-10) and sweet type (var. GT-3 and GT-7) were arranged in a randomized complete block design with four replicates at the SHS site in 2013, at the SWA site in 2013 and 2014, and at the SHC site in 2014. The selected hybrids were developed by the National Energy R&D Center for Non-food Biomass, China Agricultural University. Each plot was 36 m^2^ in size and divided into a sampling area (12 m^2^) and a harvest area (24 m^2^) for all replicates. Because soil and meteorological conditions were different each year at each experimental site, thus each year-location combination was considered an “environment” with its own specific characteristics.

Two to three seeds were sown at 0.6 × 0.2 m intervals oriented in a north–south direction using a manual hill-drop method. At the three-leaf growth stage, seedlings were manually thinned to leave one vigorous plant per hole and concurrently weeds were manually removed. All trials were carried out in accordance with good agricultural practices. However, due to concerns about extreme soil and arid conditions at the SAW site, irrigation and a higher fertilization dose were applied to the crop grown there, but not at the SHS and SHC sites. Sprinkler irrigation of approximately 30 mm of water was applied per month. Main agronomic practices and growth periods are presented in Table 3. The crop was harvested manually and harvest dates were chosen according to the timing of the killing frost.

**Table 3:** Agronomic practices in planting energy sorghum at the field experimental sites of sub-humid climate and saline-land (SHS), semi-arid climate and wasteland (SAW), and sub-humid climate and cropland (SHC).

**Agronomic practice**	**SHS site**	**SAW site**		**SHC site**
	**2013**	**2013**	**2014**	**2014**
Previous crop	Non	Non	Sorghum	Corn
Sowing date	1 May	8 May	12 May	29 April
Nitrogen fertilizer (kg N ha^−1^)	140	180	180	140
Phosphate fertilizer (kg P_2_O_5_ ha^−1^)	60	75	75	60
Potassium fertilizer (kg K_2_O ha^−1^)	60	30	30	60
Irrigation (mm)	0	150	150	0
Harvest date	28 September	3 October	24 September	15 October
Growth duration (day)	151	149	136	170

### Sample collection and measurements

On the harvest dates, tiller number was recorded for 10 hills in each plot and afterwards all aboveground plants in the harvest area of each plot were cut and weighed to estimate the fresh yield. Concurrently, 10 aboveground sorghum plants chosen randomly were harvested at the soil surface in the sampling area of each plot and were used to measure plant size (plant height and stem diameter). Next, each individual sample plant was divided into stems, leaves, and panicles, and their fresh weights were separately measured. For sampled stems, every other internode was taken from the base of each individual plant. All leaves, panicles, and sampled internodes were cut into pieces 2-to-3 cm in length and subsampled using a point-centered quarter method. Each subsample was weighed and oven-dried at 75°C until constant weight was achieved for gravimetric determination of moisture content and calculation of plant dry biomass yield.

Dried stem and leaf tissues (after panicles were removed) were ground using a Wiley mill and passed through a 0.5-mm mesh for total soluble sugar determination and through a 1-mm mesh for cellulose, hemicellulose, lignin, and ash determinations. Soluble sugar was determined in the supernatants using the anthrone-H_2_SO_4_ method and assayed using a UV–VIS spectrometer (TU-1901, Beijing Purkinje Instruments Co., Ltd., Beijing, China) according to Li et al. (2014). According to *National Renewable Energy Laboratory Analytical Procedures* (NREL LAP), cellulose, hemicellulose, and lignin were extracted using a two-step sulphuric acid hydrolysis process (Sluiter et al., 2008). Dry matter (2 g of each) was added to a 30 mL ceramic crucible to determine ash content using a muffle furnace (VULCAN 3-550, Dentsply International Inc., York, PA, USA). All chemical assays were conducted in triplicate and the average values were presented on an oven-dried basis.

### Calculations and statistical analysis

Theoretical ethanol yield (TEY) values from soluble sugar, cellulose and hemicellulose were individually calculated using the following formulas:

$$\begin{array}{l} TEY_{sugar}\;=\;total\;sugar\;content\;\times \;dry\;biomass\;\times \;F1\\ \times \;F2\times \;\frac{1000}{\rho }\\ TEY_{cellu}\;=\;cellulose\;and\;hemicellulose\;content\\ \times \;dry\;biomass\;\times \;F1\;\times \;F2\;\times \;F3\times \;F4\;\times \;\frac{1000}{\rho } \end{array}$$

Where, *TEY*_*sugar*_ represents the TEY from soluble sugar; *TEY*_*cellu*_ represents the TEY from cellulose and hemicellulose; *F*_1_ represents the coefficient of conversion factor of ethanol from sugar (0.51); *F*_2_ represents the process efficiency of ethanol from sugar (0.85); *F*_3_ represents the coefficient of 1.11 for the conversion factor of sugar from cellulose and hemicellulose; *F*_4_ represents the process efficiency of sugar from cellulose and hemicellulose (0.85); ρ represents the specific gravity of ethanol, 0.79 g mL^−1^.

Means and standard errors were calculated for the four replicates for each parameter. Two-way ANOVA was performed using the SPSS 19.0 analytical software package (IBM SPSS Inc., Chicago, IL, USA) to assess the effects of genotype, environment, and their interaction. A mean separation test was performed by using the *F*-protected least significant difference (LSD) test at 5% level of significance for each evaluated parameter. The coefficients of variation (CV) were calculated from all original determinations and defined as the ratio of the standard deviation to the mean value.

## Results and discussion

### Environmental conditions

Soil and weather variables differed considerably during the energy sorghum growing period among the three sites (Table 2). Cumulative rainfall plus irrigation was higher at the SHS (649 mm) site and SWA (580 mm in 2013 and 526 mm in 2014) site than the SHC site (390 mm) during the sorghum growing seasons (Tables 2, 3). Relative humidity, daily mean temperatures, and accumulated temperatures (≥13°C) were higher at the SHS and SHC sub-humid climate sites than the SAW semi-arid climate site, whereas cumulative sunshine hours and solar radiation varied inversely (Table 2). A maximum mean diurnal temperature difference value was observed at the SHC site, while the other sites exhibited almost no difference. Overall, the SHC site exhibited higher initial soil nutrients as compared to the marginal lands of both SHS and SAW sites.

### Effect of genotype and environment on the growth and yield of energy sorghum

Effects of variables of environment, genotype, and their interaction on all measured parameters of plant growth were significant (*p* < 0.05), with the exception of non-significant effects of genotype on tiller number and ash yield and non-significant effects of environment and genotype interaction on tiller number, stem diameter, plant moisture, lignin content, and ash yield (Table 4). The effects of the studied factors on energy sorghum growth can be ranked as environment > genotype > interaction between genotype and environment. However, an exception to this ranking was observed in only one case, for soluble sugar content and hemicellulose content, where ranking was in the order of genotype > environment > environment and genotype interaction. These findings align with those of Amaducci et al. (2004), demonstrating that year, as well as the year and genotype interaction, had significant effects on aboveground biomass yield and quality of sweet and biomass sorghum. Furthermore, Zhao et al. (2009) concluded that effects of year and genotype on biomass, carbohydrates, and ethanol yield were highly significant (*p* < 0.001) and that differences among various years were ultimately attributed to variations in environmental conditions.

**Table 4:** Combined analyses of variance (*F*-value) for morphological and chemical characteristics of energy sorghum evaluated for four environments under field conditions.

**Parameter**	**Environment (*df* = 3)**	**Genotype (*df* = 4)**	**Environment × Genotype (*df* = 12)**
Tiller number	15.6^***^	2.3^ns^	0.9^ns^
Plant height	631.0^***^	79.9^***^	2.7^**^
Stem diameter	24.9^***^	9.7^***^	1.4^ns^
Plant moisture	139.8^***^	6.7^***^	0.9^ns^
Biomass yield	231.3^***^	4.9^**^	3.2^**^
Soluble sugar content	21.0^***^	29.7^***^	4.5^***^
Soluble sugar yield	195.3^***^	12.3^***^	3.8^***^
Cellulose content	54.2^***^	28.4^***^	3.6^**^
Cellulose yield	291.0^***^	11.1^***^	5.8^***^
Hemicellulose content	20.6^***^	42.6^***^	7.8^***^
Hemicellulose yield	277.6^***^	13.5^***^	7.0^***^
Lignin content	50.9^***^	3.7^*^	1.4^ns^
Lignin yield	205.9^***^	9.9^***^	6.7^***^
Ash content	85.3^***^	10.5^***^	2.8^**^
Ash yield	105.7^***^	1.5^ns^	1.6^ns^
Theoretical ethanol yield	301.3^***^	4.7^**^	2.8^**^

ns Non-significant effects;

* Significant effect at p < 0.05 level;

** Significant effect at p < 0.01 level;

*** *Significant effect at p < 0.001 level*.

### Tiller number, plant size, and moisture content

Tiller number, plant size, and moisture content showed significant differences (*p* < 0.05) among the experimental sites and the energy sorghum hybrids (Tables 5, 6). Averaged across all the hybrids, both SAW, and SHS sites produced plants with smaller size, higher tiller number, and higher plant moisture content in comparison with plants of the SHC site (Table 5), whereas each of these parameters was lower for sorghum at the SAW site vs. the SHS site. Moreover, biomass type hybrids exhibited larger plant sizes than sweet type hybrids did, whereas tiller number and plant moisture were higher in sweet type hybrids (Table 6).

**Table 5:** Energy sorghum characteristics for performance at the experimental sites of sub-humid climate and saline-land (SHS), semi-arid climate and wasteland (SAW), and sub-humid climate and cropland (SHC).

**Character**	**SHS site**	**SAW site**		**SHC site**
	**2013**	**2013**	**2014**	**2014**
Plant height (cm)	444 a	258 b	227 c	444 a
Stem diameter (mm)	17.3 a	15.1 b	14.9 c	17.0 a
Tiller number (no.)	0.5 a	0.2 b	0.2 b	0.2 b
Plant moisture (%)	83.6 a	66.6 c	72.9 b	65.3 c
Soluble sugar content (g kg^−1^)	171 b	111 c	118 c	201 a
Soluble sugar yield (t ha^−1^)	1.3 b	0.7 c	0.7 c	5.9 a
Cellulose content (g kg^−1^)	384 a	345 b	298 d	330 c
Cellulose yield (t ha^−1^)	3.2 b	2.0 c	1.8 c	10.0 a
Hemicellulose content (g kg^−1^)	238 a	239 a	226 b	206 c
Hemicellulose yield (t ha^−1^)	2.0 b	1.4 c	1.4 c	6.3 a
Lignin content (g kg^−1^)	173 b	247 a	155 b	158 b
Lignin yield (t ha^−1^)	1.5 b	1.5 b	0.9 c	4.8 a
Ash content (g kg^−1^)	40 c	56 b	61 a	30 d
Ash yield (t ha^−1^)	0.3 b	0.3 b	0.3 b	0.9 a

*Different small letters within a row indicate significant differences at p < 0.05*.

**Table 6:** Plant size, tiller number, and plant moisture of the energy sorghum hybrids averaged across the experimental sites of sub-humid climate and saline-land (SHS), semi-arid climate and wasteland (SAW), and sub-humid climate and cropland (SHC).

**Type**	**Hybrid**	**Plant height (cm)**	**Stem diameter (mm)**	**Tiller number (no.)**	**Plant moisture (%)**
Biomass sorghum	GN-2	363 b	16.2 ab	0.2 ab	71.7 b
	GN-4	363 b	17.0 a	0.2 b	71.1 b
	GN-10	379 a	16.2 ab	0.3 a	68.7 c
	Average	368	16.5	0.2	70.5
Sweet sorghum	GT-3	312 c	15.8 b	0.3 a	74.7 a
	GT-7	262 d	14.7 c	0.3 ab	71.3 b
	Average	287	15.2	0.3	73.0

*Regardless of sorghum type, different small letters within a column indicate significant differences at p < 0.05*.

In general, larger plant size is partially responsible for the highest observed biomass yield at the SHC site and showed a significantly positive correlation (*p* < 0.01) with biomass yield (*r* = 0.663 for plant height and *r* = 0.471 for stem diameter). In addition, the longer growth period at the SHC site also contributed to higher biomass yield, as did lower tiller number, as observed previously (Huang et al., 2013). Moreover, Ao et al. (2010) demonstrated that low tiller number values can facilitate synchronous harvest by promoting uniformity of plant characteristics, ensuring a more efficient use of horizontal space. Furthermore, low plant moisture of biomass sorghum is very conducive to rapid drying for facilitated transportation and storage (Zegada-Lizarazu and Monti, 2012; Iqbal et al., 2017).

### Biomass yield and stem, leaf, panicle partitioning

**Figure 1:**
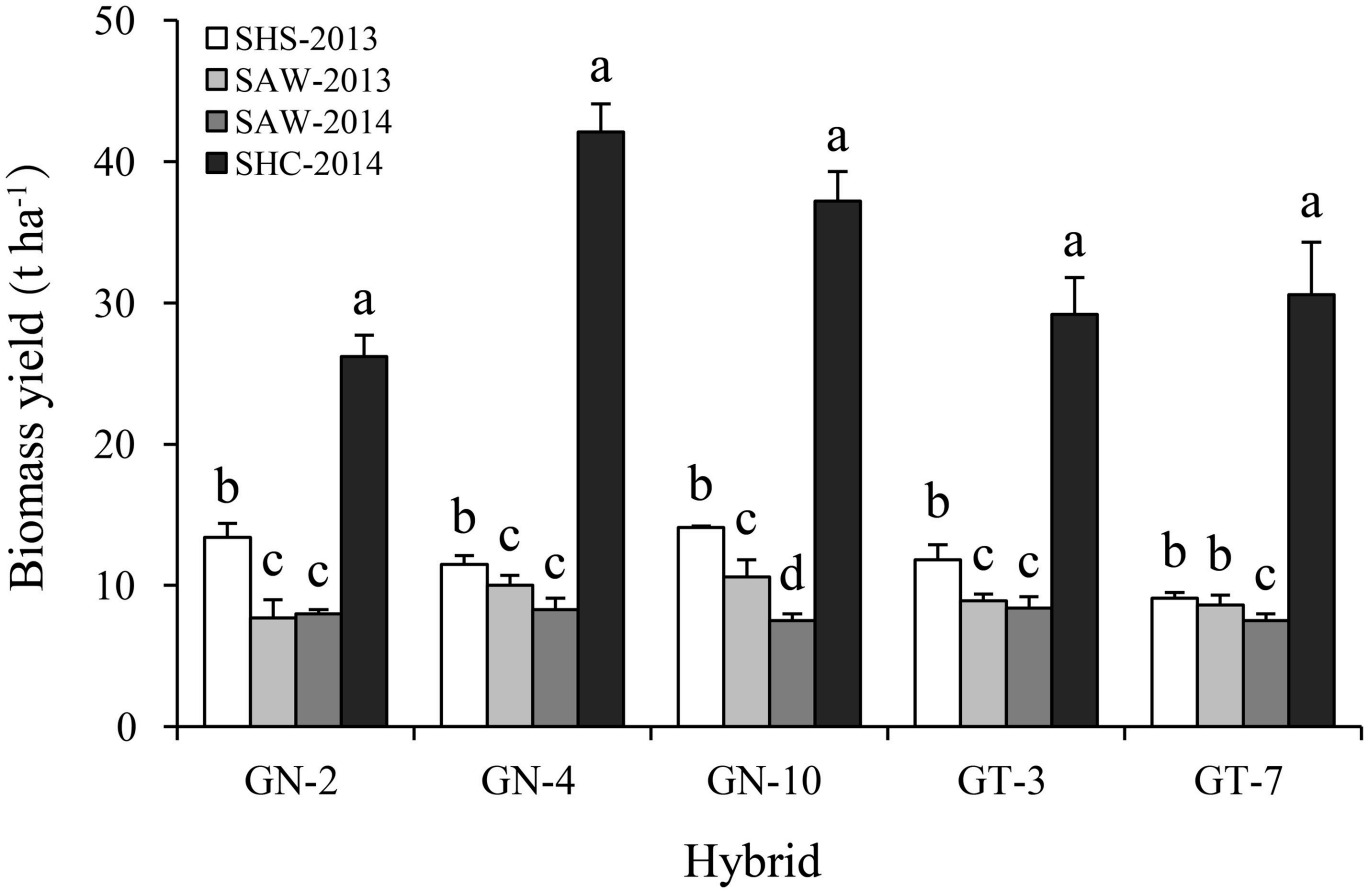
Biomass yield of energy sorghum hybrids at sites of sub-humid climate and saline-land (SHS), semi-arid climate and wasteland (SAW), and sub-humid climate and cropland (SHC) in 2013 and 2014. The different small letters indicate significant differences within environments for each hybrid at the *p* < 0.05 level. Vertical bars represent standard errors.

Obviously, biomass yields averaged across all energy sorghum hybrids grown at either the SAW site (8.6 t ha^−1^ for average of 2013 and 2014) or SHS site (12.2 t ha^−1^) were statistically (*p* < 0.01) lower compared to average yield for hybrids grown at the SHC site (32.6 t ha^−1^) (Figure 1). However, energy sorghum at the SHS site showed a significantly (*p* < 0.05) higher biomass yield (41.9%) than at the SAW site. In general, salt stress at the SHS site or infertile soil coupled with higher evaporation probably leading to soil water stress at the SAW site decrease biomass yield relative to the regular cropland conditions at the SHC site. The dramatic differences in biomass yield at different sites in this study could be attributed to considerable diversity in environmental factors, such as climate (precipitation, temperature, and evaporation) and soil type and fertility. Tang et al. (2015) demonstrated that precipitation and soil organic matter were key environmental factors influencing biomass yield of sweet sorghum. Meanwhile, high altitude also caused a decline in sweet sorghum production due to a lower temperature (Li and Feng, 2013). Previous studies confirmed that well-timed irrigation could considerably improve biomass yield (Mastrorilli et al., 1995; Dercas and Liakatas, 2007). Habyarimana et al. (2004) demonstrated that higher aboveground biomass yield of sorghum ranged from 33 to 51 t ha^−1^ under irrigation than that of 20–29 t ha^−1^ under rain-fed conditions in the Mediterranean region. Cosentino et al. (2012) reported that sweet sorghum produced 7.5 t ha^−1^ of dry matter with 80 mm irrigation vs. 21.1 t ha^−1^ with 334 mm irrigation under semi-arid conditions.

While lower than cropland biomass yields, yields on marginal lands studied here were comparable to yields of previous field studies conducted under similar environmental conditions. For instance, Ameen et al. (2017) and Fu et al. (2016) measured biomass yield of energy sorghum fluctuating from 4.9 to 14.2 t ha^−1^ on a sandy loam soil of marginal land in Inner Mongolia. A recent study by Tang et al. (2018) reported that energy sorghum exhibited a good biomass yield (6.1–9.2 t ha^−1^) due to its superior adaptability to abandoned marginal land. In another study conducted in northern Greece, significantly lower sweet sorghum biomass yield (13.7 t ha^−1^) was observed in soil with high salinity (Vasilakoglou et al., 2011).

Averaged across hybrids, biomass type sorghum exhibited significantly (*p* < 0.05) higher biomass yield (17.3 t ha^−1^) than sweet type (14.7 t ha^−1^), with a particularly greater difference in biomass type vs. sweet type yields at the SHC site (34.5 vs. 29.9 t ha^−1^, respectively) (Figure 1). Thus, biomass type sorghum holds a promising future for energy generation due to its higher biomass production compared to that of sweet type sorghum in this study. With regard to two type's hybrids across all sites, biomass type hybrid GN-10 showed the highest average biomass yield (17.4 t ha^−1^) and is particularly well-adapted to adverse environmental conditions such as water deficits, salinity, and alkalinity. Considering only biomass yield performance as the major priority, biomass type hybrid GN-4 demonstrated a very high biomass yield of 42.1 t ha^−1^ after growth on cropland (but not on marginal land) under sub-humid climate conditions at the SHC site. Other research groups have also achieved successful growth of energy sorghum in sub-humid climate conditions, including Gnansounou et al. (2005) who reported that sorghum for energy purpose was well adapted to temperate sub-humid climates, and Zhao et al. (2009), who reported that sweet sorghum exhibited a high biomass yield of 35.2 t ha^−1^ after 40 days following anthesis under sub-humid climate conditions.

**Figure 2:**
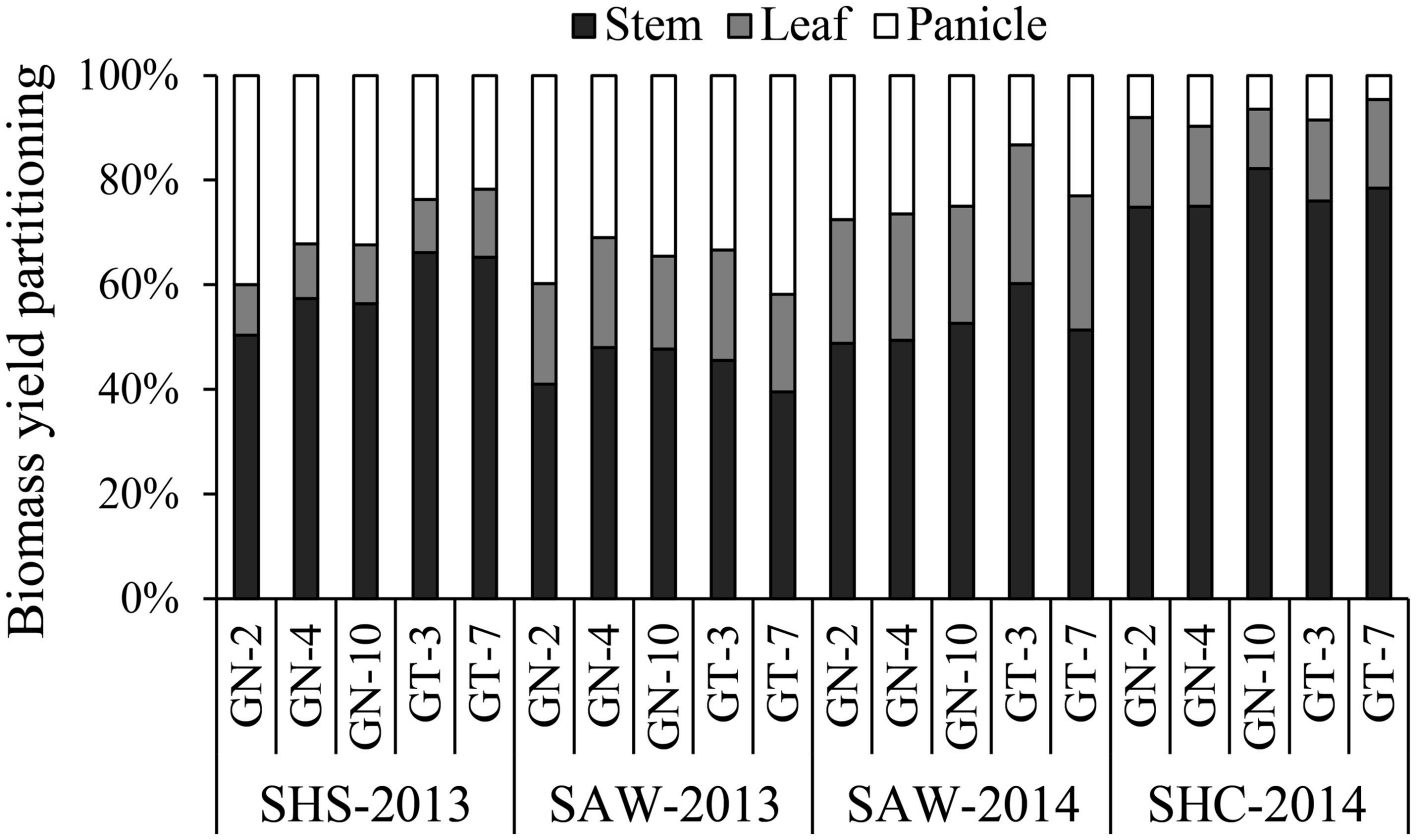
Dry biomass yield partitioning of energy sorghum hybrids as determined by the weight fractions of stem, leaf, and panicle for different experimental sites of sub-humid climate and saline-land (SHS), semi-arid climate and wasteland (SAW), and sub-humid climate and cropland (SHC) in 2013 and 2014.

Biomass yield partitioning across all the hybrids showed that stem weight represented the highest proportion (74.8–82.3%) of total dry biomass at the SHC site to the values at the SHS site (50.4–66.1%) and SAW site (39.5–60.2%). Panicle biomass was found to be significantly (*p* < 0.05) the lowest proportion of total biomass, ranging between 4.6 and 9.7% at SHC site (Figure 2). Notably, sweet type sorghum hybrids exhibited higher overall values of stem (60.3 vs. 57.0%) and leaf biomass yield (18.4 vs. 16.9%) than biomass type hybrids.

### Chemical components

**Figure 3:**
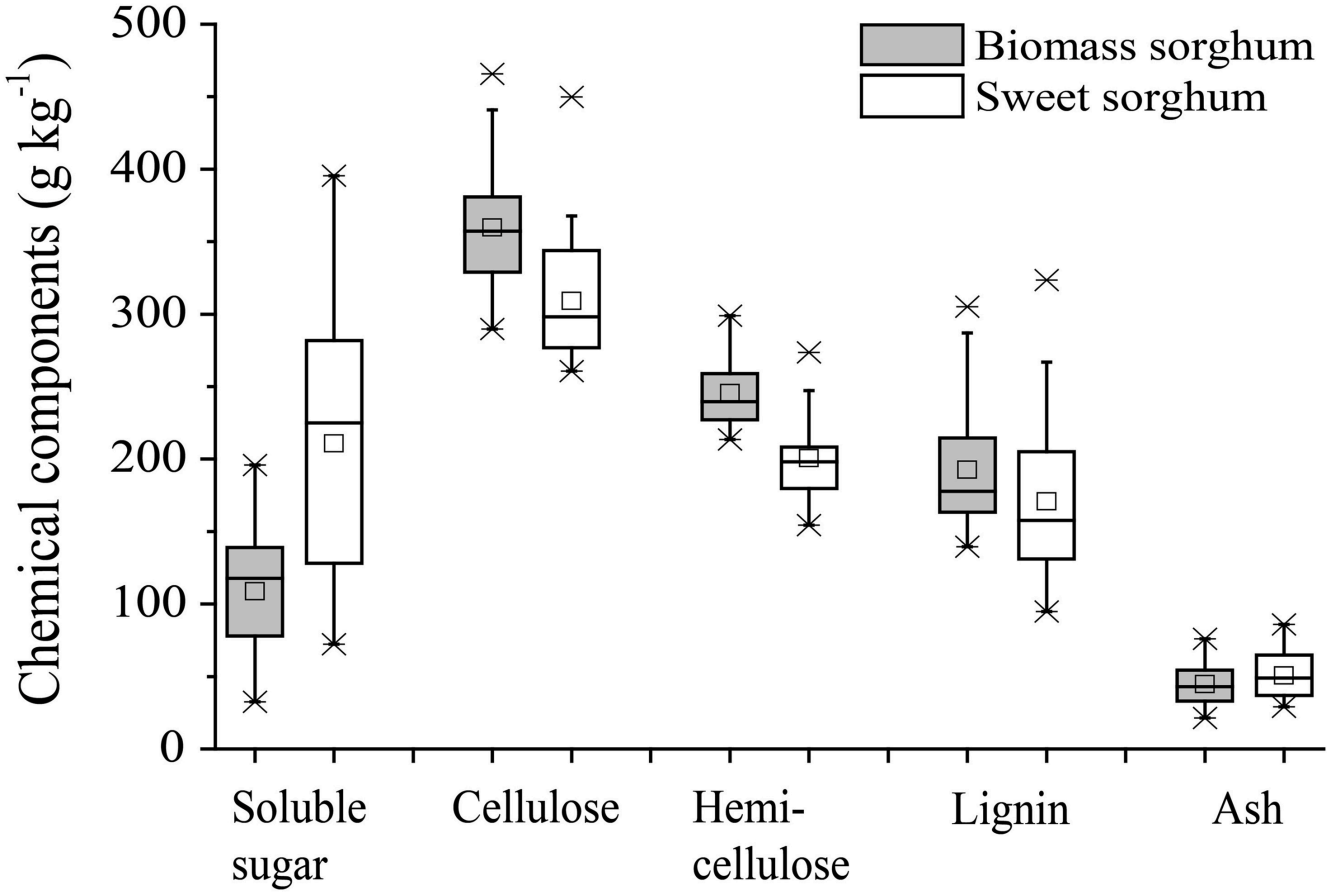
Variations in chemical components of whole plants of biomass and sweet sorghum at the experimental sites of sub-humid climate and saline-land (SHS), semi-arid climate and wasteland (SAW), and sub-humid climate and cropland (SHC).

Energy sorghum chemical components were significantly affected by environment and sorghum genotype. Across all sites, a relatively high coefficient of variation (CV) was observed for soluble sugar (34.5%), lignin (26.1%), and ash (33.7%), whereas cellulose and hemicellulose content exhibited relatively lower variability, with CV values of 13.4 and 10.4%, respectively (Figure 3). Previous studies reported that sucrose, cellulose, hemicellulose, and ash content varied significantly with locations, while lignin content remained relatively constant (Amaducci et al., 2004; Singh et al., 2012; Wei et al., 2014). After comparison of the three sites in this study (Table 5), we determined that under sub-humid climate conditions, the SHC site was most conducive to obtaining ideal soluble sugar content, while the SHS site was conducive to obtaining higher cellulose and hemicellulose content. However, higher content of lignin and ash observed for sorghum from the SAW site demonstrated that undesirable components of cellulosic materials may easily be produced on sandy wasteland under the water deficit conditions of a semi-arid region. Therefore, energy sorghum cultivated in a sub-humid climate is recommended instead for use as solid biofuel feedstock for thermal utilization, due to its lower ash content (Pannacci and Bartolini, 2016).

Meanwhile, yield of all chemical components in aboveground plants was significantly (*p* < 0.05) higher at the SHC site (Table 5), due to significantly higher overall biomass production. In particular, the yields of three desirable components (soluble sugar, cellulose, and hemicellulose) on marginal lands were 4.5–8.4 times lower at the SAW site (average of 2013 and 2014) than the SHC site and 3.2–4.5 times lower at the SHS site than at the SHC site. On the one hand, water supply and normal agricultural land for conservation tillage positively affected cellulosic biomass production (Rocateli et al., 2012). On the other hand, for energy purpose total cellulosic biomass yield is much more important than cellulosic biomass quality for selection of the optimal energy sorghum hybrids.

As an additional consideration, biomass type sorghum is predominantly composed of structural carbohydrates (cellulose and hemicellulose) (Figure 3). It exhibited significantly (*p* < 0.01) higher (by 27.0–34.8%) yields of cellulose, hemicellulose, and lignin than the sweet type. However, reverse trends were observed for yields of soluble sugars and ash, which were lower (by 87.5 and 20%, respectively) for biomass type sorghum when averaged across all hybrids and sites (Table 7). Moreover, hybrid GN-10 biomass type sorghum exhibited higher contents of desirable components (including soluble sugar, cellulose, and hemicellulose) and lower contents of lignin and ash in aboveground plants, while producing the highest yields (10.5 t ha^−1^) of the first three aforementioned components across all sites. Between the two hybrids of sweet type sorghum analyzed, GT-7 produced higher yields of all chemical components except for the yield of soluble sugar.

**Table 7:** Content and yield of chemical components in plants of different energy sorghum hybrids averaged across the experimental sites of sub-humid climate and saline-land (SHS), semi-arid climate and wasteland (SAW), and sub-humid climate and cropland (SHC).

**Type**	**Hybrid**	**Soluble sugar**	**Cellulose**	**Hemicellulose**	**Lignin**	**Ash**
Content	Biomass sorghum					
(g kg^−1^)	GN-2	101 b	359 a	246 a	196 a	49 ab
	GN-4	111 b	358 a	243 a	198 a	46 b
	GN-10	114 b	363 a	247 a	184 ab	39 c
	Average	109	360	245	193	42
	Sweet sorghum					
	GT-3	214 a	304 b	200 b	169 b	47 b
	GT-7	207 a	315 b	201 b	172 b	54 a
	Average	210	310	201	171	50
Yield	Biomass sorghum					
(t ha^−1^)	GN-2	1.3 b	3.9 a	2.6 a	2.0 b	0.4 b
	GN-4	1.8 b	4.9 a	3.2 a	2.6 a	0.5 ab
	GN-10	1.8 b	5.3 a	3.4 a	2.6 a	0.5 b
	Average	1.6	4.7	3.1	2.4	0.5
	Sweet sorghum					
	GT-3	3.1 a	3.6 b	2.2 b	1.8 b	0.5 ab
	GT-7	2.9 a	3.9 b	2.5 b	1.9 b	0.6 a
	Average	3.0	3.7	2.3	1.8	0.6

*Regardless of sorghum type, different small letters within a column indicate significant differences at p < 0.05*.

**Figure 4:**
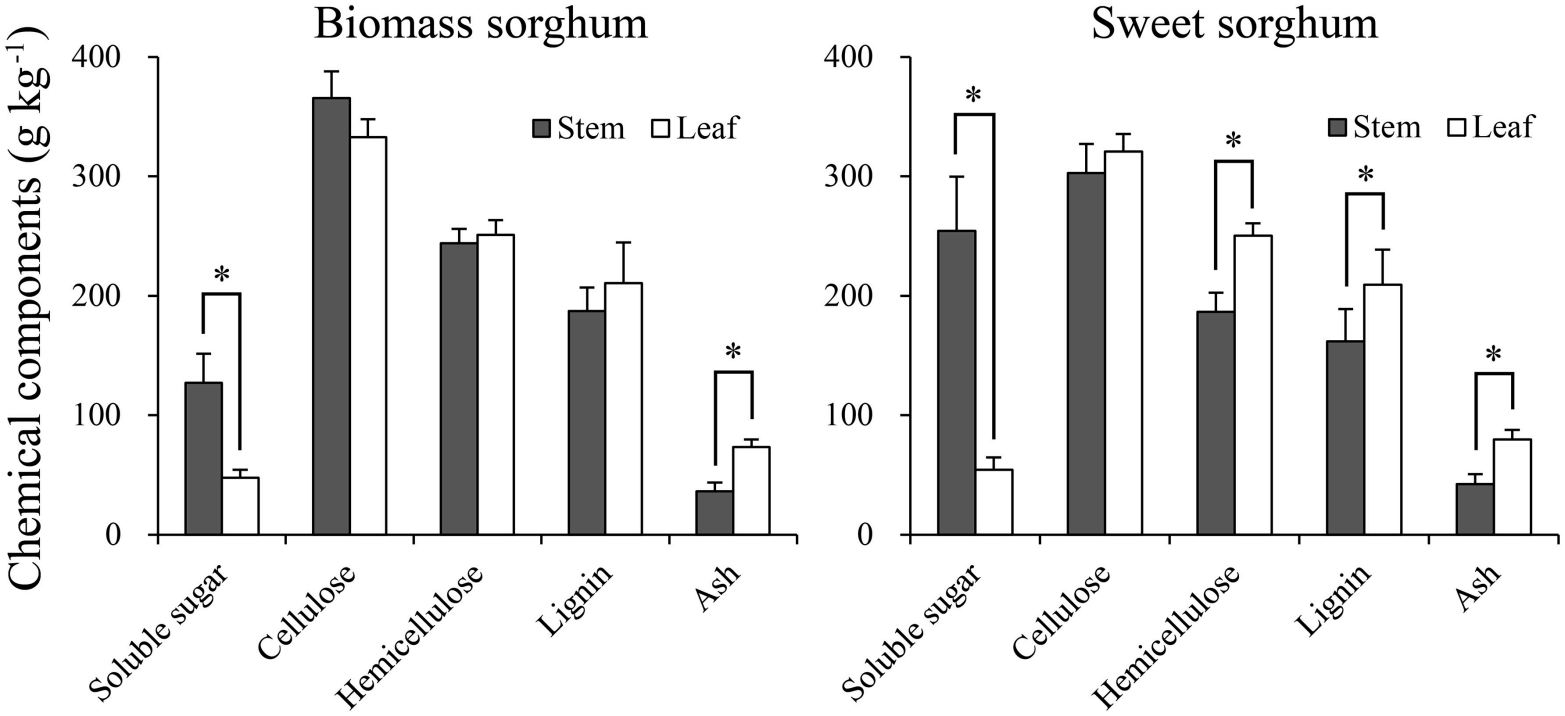
Chemical components partitioning of biomass and sweet sorghum into stem and leaf averaged across all hybrids used at the experimental sites of sub-humid climate and saline-land (SHS), semi-arid climate and wasteland (SAW), and sub-humid climate and cropland (SHC). Asterisks indicate significant differences within stem and leaf for each component at the *p* < 0.05 level. Vertical bars represent standard errors.

With regard to components partitioning, soluble sugar in stem was significantly (*p* < 0.05) higher (3.7 times) than in leaf when averaged across sites and hybrids (Figure 4). Moreover, ratios of components in leaf vs. stem were as follows: hemicellulose content (1.2 times), lignin (1.2 times), and ash (1.9 times). However, while cellulose content was 9.9% higher in stem than in leaf of biomass type sorghum, cellulose was 6.0% lower in stem than leaf of sweet type sorghum. These findings agreed with results of Zhao et al. (2009) and Monti et al. (2008).

### Theoretical ethanol yield (TEY)

**Figure 5:**
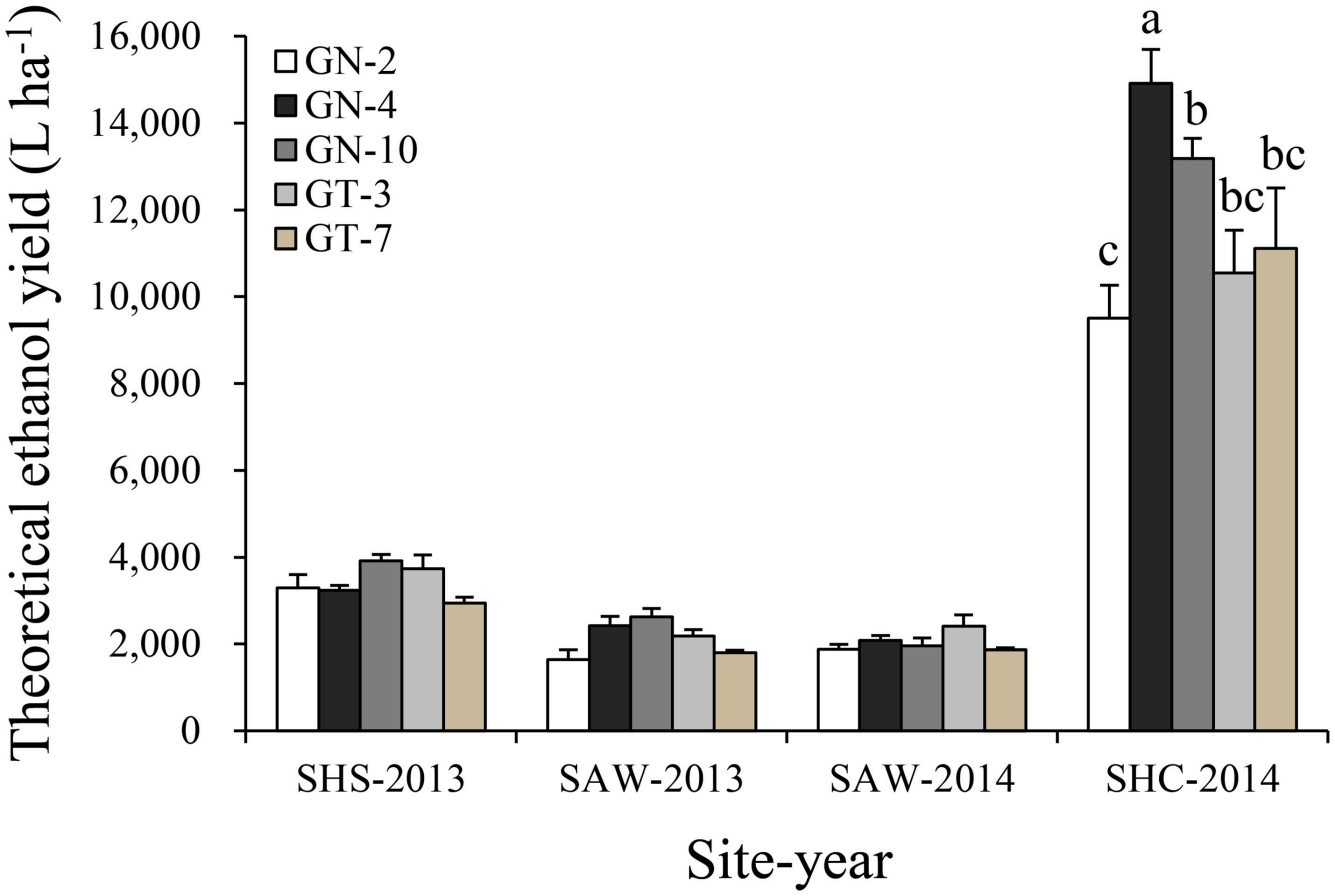
Theoretical ethanol yield of energy sorghum hybrids at sites of sub-humid climate and saline-land (SHS), semi-arid climate and wasteland (SAW), and sub-humid climate and cropland (SHC) in 2013 and 2014. The different small letters indicate significant differences within each hybrid and each site at the *p* < 0.05 level. The vertical bars indicate standard errors.

**Figure 6:**
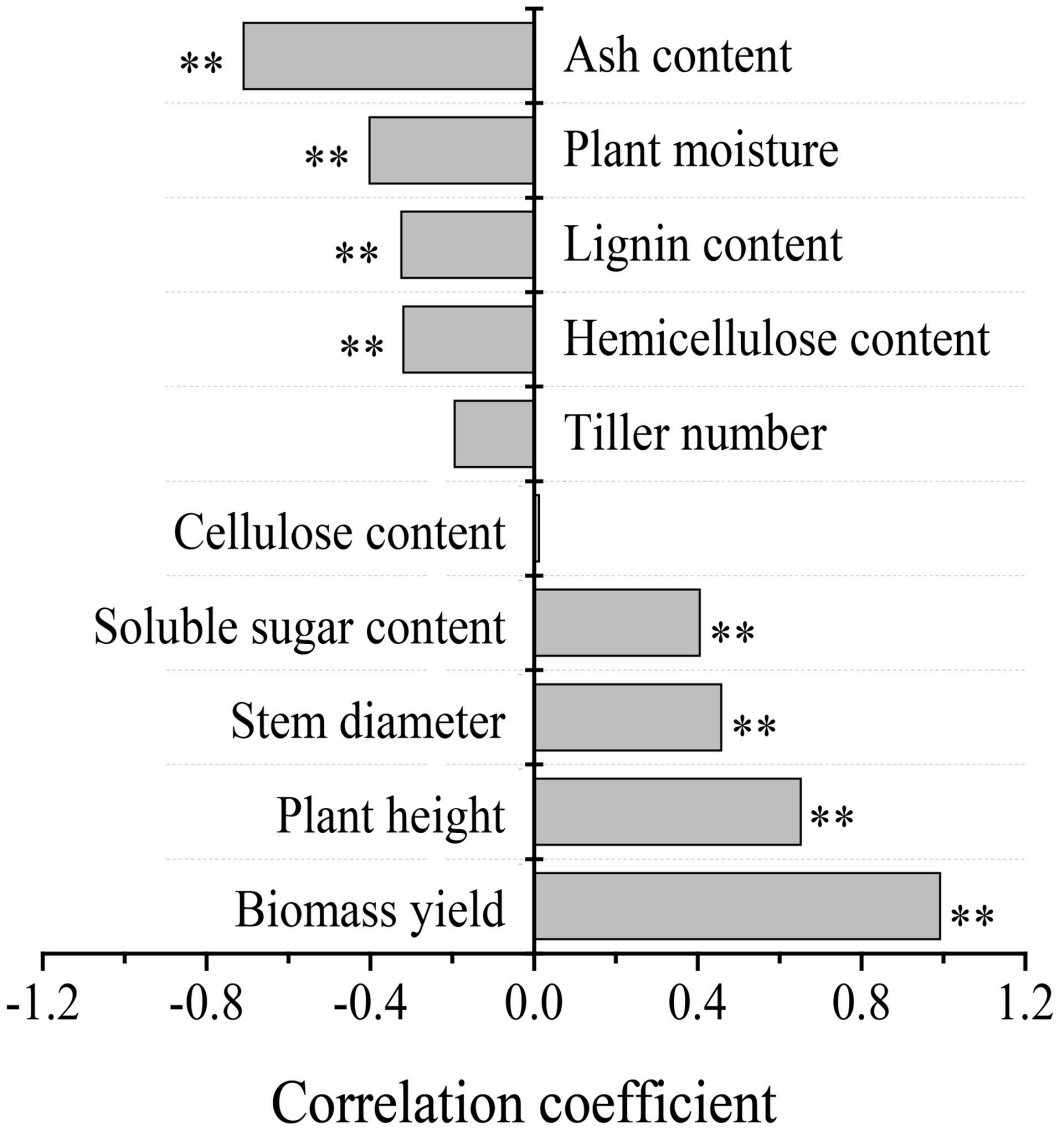
Analysis of correlation between theoretical ethanol yield and related growth, yield and quality parameters of energy sorghum production. Two asterisks indicate significant correlations at the *p* < 0.01 level.

High TEY yield mirrored biomass yield in this study; a TEY >11,853 L ha^−1^ was observed at the SHC site, which produced 3.5 times (*p* < 0.05) higher ethanol yield than that observed at the SHS site (3,425 L ha^−1^) and 5.7 times greater yield than at the SAW site (2,091 L ha^−1^, averaged of 2013 and 2014) (Figure 5). Furthermore, correlation analysis of biomass yield, plant height, stem diameter, and soluble sugar content showed significantly (*p* < 0.01) positive correlations with TEY; however, the content of ash, lignin, and hemicellulose and plant moisture were negatively correlated with TEY (*p* < 0.01, Figure 6). However, tiller number and cellulose content were not significantly correlated with TEY, which indicates that both parameters did not affect ethanol production.

The TEY values for marginal lands including saline-land and dry wasteland reflected severely reduced potential ethanol production relative to cropland. According to Fu et al. (2016), sweet sorghum grown on sandy loam soil exhibited TEY of 2,491 L ha^−1^ from stalk of the crop in a semi-arid region in northern China. Vasilakoglou et al. (2011) reported an ethanol yield of 2,623 L ha^−1^ from sweet sorghum on land with salinity 6.9 dS m^−1^. Wortmann et al. (2010) reported a potential ethanol yield of 2,211 L ha^−1^ using biomass of sweet sorghum grown at seven dryland site-years in a semi-arid region. However, much higher ethanol yield on cropland under sub-humid climate conditions at Missouri, USA, was reported by Houx and Fritschi (2013) and Maw et al. (2016), indicating that sweet sorghum can achieve TEY values of 5,000–7,488 L ha^−1^. Moreover, Zhao et al. (2012) reported that high-yielding sweet sorghum cultivars provided the highest ethanol yield potential ranging between 9,097 and 10,803 L ha^−1^ from sugar, starch, cellulose, and hemicellulose, on a cropland geographically near to the SHC site of this study. The reason for the large gap of ethanol potential from sweet sorghum between marginal land and cropland would probably be the variations in temperature, precipitation, evaporation, soil fertility, and management practices, which could substantially impact crop biomass yield and components.

In this study, the biomass type sorghum exhibited a higher TEY magnitude compared with sweet type sorghum (5,056 vs. 4,578 L ha^−1^) averaged across all sites. In particular, hybrid GN-10 biomass type sorghum produced the highest TEY (5,423 L ha^−1^, Figure 5), which was 34.1% higher than the lowest TEY observed for hybrid GN-2. Hybrid GN-4 produced significantly (*p* < 0.05) highest ethanol yield at the SHC site relative to the other hybrids, exhibiting the highest value of 14,913 L ha^−1^.

### Future perspectives

In this study, energy sorghum grown on marginal lands exhibited a much lower ethanol potential than that on cropland, indicating a considerable lower possibility for being used as commercial feedstock production due to environmental stresses and an additional input. At a saline-alkali site Wuyuan in northern China, sweet sorghum showed negative economic performance, whereas the reference crops maize and sunflower exhibited relatively high positive benefit (Liu et al., 2015). For sustainable commercial energy sorghum production, marginal lands with relatively low environmental stresses should be selected and stress-resistant plantation technologies should be developed. It is important to screen stress-resistant varieties with genetic improvement strategy and establish efficient crop production systems with conservation tillage (Xie, 2012). Favorable policy is particularly of significance in non-food biofuel development. Economic incentives including specific capital subsidies, low-cost financing, tax incentives and R&D funding should be established to promote non-food energy crop production in marginal lands.

## Conclusions

This study revealed environmental stress affecting biomass yield to guide future development of promising sorghum hybrids adapted to growth on marginal lands. As part of a larger sustainable agro-industrial framework, biomass type sorghum feedstock should be encouraged for industrial scale ethanol production due to its high productivity, adaptation to marginal growth conditions, and desirable qualities that facilitate efficient conversion of its biomass to ethanol. In particular, hybrid biomass type GN-10 possesses all of these attributes, while being especially well-adapted to growth in adverse environmental conditions such as water deficits, salinity, and alkalinity. However, from an output point of view, biomass type hybrid GN-4 achieved the highest values of biomass yield (42.1 t ha^−1^) and TEY (14,913 L ha^−1^) on cropland in a sub-humid climate. Ultimately, lower ethanol potential of energy sorghum grown on marginal land reflected a lower possibility for commercial feedstock supply than that grown on regular cropland. As well, screening suitable varieties could improve energy sorghum growth and chemical components for ethanol production.

## Author contributions

CT: Analyzed the data, made tables and figures, and drafted the manuscript. SL and ML: Performed the laboratory experiments and collected the data. GX: Designed and supervised the field and laboratory work and finalized the manuscript.

### Conflict of interest statement

The authors declare that the research was conducted in the absence of any commercial or financial relationships that could be construed as a potential conflict of interest.
